# Matrimid‐Derived Asymmetric Carbon Molecular Sieve Hollow Fibers With Engineered Ultramicropores for Precise Helium Separation

**DOI:** 10.1002/anie.6271309

**Published:** 2026-03-23

**Authors:** Zhongyun Liu, Yuhe Cao, Ryan P. Lively, William J. Koros

**Affiliations:** ^1^ School of Chemical & Biomolecular Engineering Georgia Institute of Technology Atlanta Georgia USA; ^2^ Key Lab of Functional Polymers For Sustainability of Jiangsu, School of Energy and Environment Southeast University Nanjing Jiangsu P.R. China; ^3^ Department of Civil, Environmental and Construction Engineering The University of Texas at El Paso El Paso Texas USA

**Keywords:** carbon molecular sieve hollow fiber, He/CH_4_ separation, hyperaging, ultramicropore tuning

## Abstract

Helium/methane (He/CH_4_) separation is of strategic importance for energy and industrial applications, yet it remains technically challenging due to the need to simultaneously achieve ultrahigh selectivity and helium productivity. In this work, we report ultraselective Matrimid‐derived asymmetric carbon molecular sieve (CMS) hollow fibers in which ultramicropore and fiber geometry are deliberately co‐engineered to enable precise He/CH_4_ separation with high He productivity at the module level. We showed that pyrolysis temperature tuning tightens ångström‐scale ultramicropores and enhances He/CH_4_ discrimination; while a targeted post‐pyrolysis hyperaging enables selective refining of the ultramicropores, thereby offering high He permeance with exceptional He/CH_4_ selectivity. For a 5/95 He/CH_4_ mixed gas feed, the hyperaged CMS‐700 hollow fibers achieve permeate helium purities of up to 98.6% with He/CH_4_ selectivities exceeding 1300 and a stable He permeance of approximately 26 GPU. Beyond this material achievement, fiber geometry optimization through reduction of the outer diameter was achieved to increase the packable membrane area without compromising mechanical integrity or intrinsic separation performance, leading to enhanced module‐level He productivity. This integrated co‐engineering strategy provides an energy‐efficient and industrially viable platform for He recovery and is readily extendable to other challenging small/large gas‐pair separations.

## Graphical abstract

Matrimid‐derived asymmetric carbon molecular sieve (CMS) hollow fibers enable ultraselective helium/methane (He/CH_4_) separation. Post‐pyrolysis hyperaging selectively tightens larger ultramicropores, enhancing He/CH_4_ discrimination while maintaining high He permeance. Reducing CMS fiber outer diameter increases packable area and module throughput, coupling high selectivity with high module‐level He productivity.

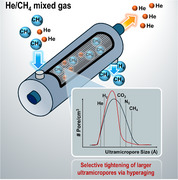

Efficient separation of helium (He) from methane (CH_4_) is of critical strategic and economic importance. Helium is a critical yet non‐renewable resource that plays essential roles across diverse sectors [1], including scientific research, aerospace, medical technology, and high‐tech manufacturing. Currently, the primary source of helium is natural gas, from which it is recovered through energy‐intensive cryogenic distillation with high carbon emissions [2]. These challenges have driven increasing interest in energy‐efficient membrane‐based separation technologies for such separations [3, 4, 5, 6, 7]. Considering the low helium content in natural gas (< 0.1–9.8 mol%) [8, 9, 10] and the high‐purity requirements for its end‐use applications, developing ultraselective membranes capable of precise He/CH_4_ discrimination is crucial for sustainable helium recovery and long‐term supply security.

Carbon molecular sieve (CMS) membranes combine outstanding separation performance with scalable processability, making them a promising platform for advanced gas separations [11, 12]. Specifically, the tunable ångström‐scale ultramicropores in CMS enable exceptional molecular discrimination based on subtle differences in gas molecule size and shape. Moreover, CMS membranes can be fabricated as mechanically robust hollow fibers, enabling the construction of high surface‐to‐volume and compact modular devices suitable for large‐scale industrial applications [13, 14]. In previous work, Matrimid‐derived CMS hollow fibers have been reported for CO_2_/CH_4_ [15, 16, 17], N_2_/CH_4_ [18], and light hydrocarbon separations [19, 20]; however, their application to helium purification has not been explored. In this work, we demonstrate that ultraselective helium separation from He/CH_4_ mixtures can be achieved using Matrimid‐derived CMS hollow fibers with engineered ultramicropores tailored for He/CH_4_ discrimination. Controlled pyrolysis of vinyltrimethoxysilane (VTMS)‐treated precursors [13, 14] followed by targeted hyperaging selectively tightens larger ultramicropores to enable an uncommon combination of ultrahigh He/CH_4_ selectivity and high He permeance. Furthermore, reducing fiber outer diameter serves as a module‐level relevant feature to enhance helium productivity without compromising intrinsic separation performance. Together, this work establishes commercially available Matrimid‐derived CMS hollow fibers as a scalable and previously unexplored platform for helium recovery, extending CMS technology beyond prior state‐of‐the‐art separations.

To prepare high‐performance CMS hollow fibers, spinning defect‐free, thin‐skinned hollow fiber precursors is important. In our work, Matrimid hollow fiber precursors were prepared via a dry‐jet wet‐quench spinning process [21, 22], and detailed spinning conditions (Table S1) are provided in the Supporting Information. The resulting Matrimid fibers exhibited an asymmetric morphology (Figures 1a and S1) comprising a thin selective skin layer and a porous substructure. The O_2_/N_2_ selectivity of the Matrimid precursors with a take‐up rate of 50 m min^−1^ was measured to be 7.2, slightly higher than that of Matrimid dense films (6.7), confirming the prepared fiber precursors are defect‐free (Table S2). Prior to pyrolysis, a sol–gel VTMS treatment was applied to prevent excessive collapse of the porous substructure during carbonization [17, 23]. As shown in Figure 1b–d, all the resulting CMS hollow fibers retained their asymmetric morphology, which is preferred for minimizing mass‐transfer resistance in CMS fiber walls to enable high gas permeance. In contrast, in the absence of VTMS treatment, the porous substructure collapsed completely, yielding a fully densified CMS fiber wall (Figure S2).

**FIGURE 1:**
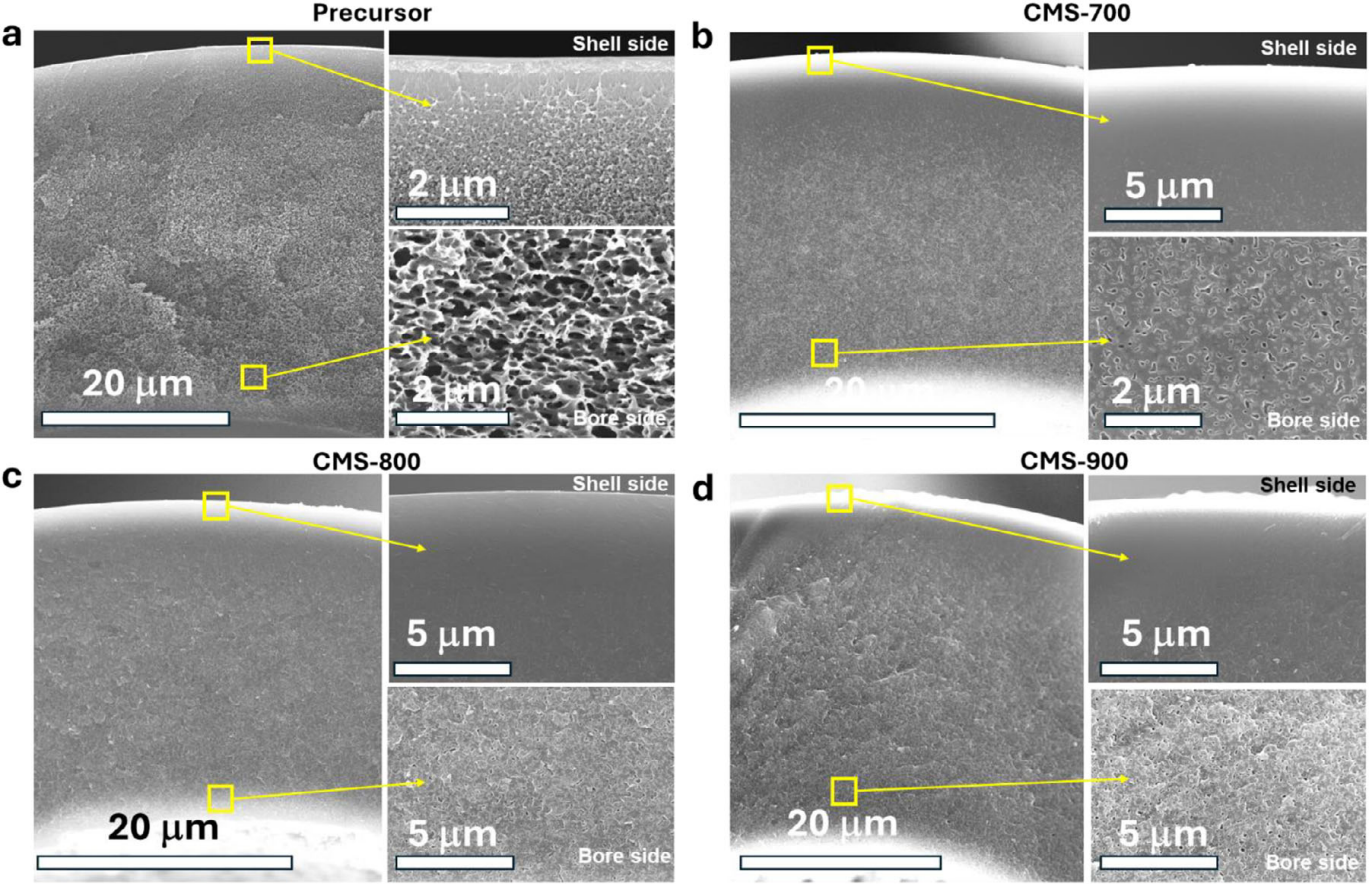
SEM images of Matrimid hollow fiber precursor (a) and CMS fibers pyrolyzed under different temperatures (b–d). Without VTMS, the morphology in (b–d) looks like the densified cases at the shell side.

Beyond optimizing the macroscopic morphology of CMS hollow fibers, the ultramicropore structure was also tuned by optimizing the pyrolysis temperature for He/CH_4_ separation. Pure gas permeation measurements of the prepared CMS hollow fibers revealed a clear decrease in permeance with increasing gas kinetic diameter, confirming the molecular sieving nature of the CMS membranes (Figure 2a). With increasing pyrolysis temperature from 700 to 900°C, the overall gas permeance decreased while the larger CH_4_ molecules showed a more pronounced reduction, indicating the tightening of ultramicropores at higher temperatures that restrict CH_4_ diffusion and thereby increase the He/CH_4_ selectivity (Figure 2a,b). In contrast to He/CH_4_ separation, elevating the pyrolysis temperature over this range showed little effect on He/N_2_ selectivity (Figure 2c), showing that the tightened ultramicropores still allow the permeation of slimmer N_2_ molecules. The He/CH_4_ separation performance of the CMS fibers was evaluated using a 5/95 He/CH_4_ mixture feed at 35°C and a total feed pressure of 100 psia.

**FIGURE 2:**
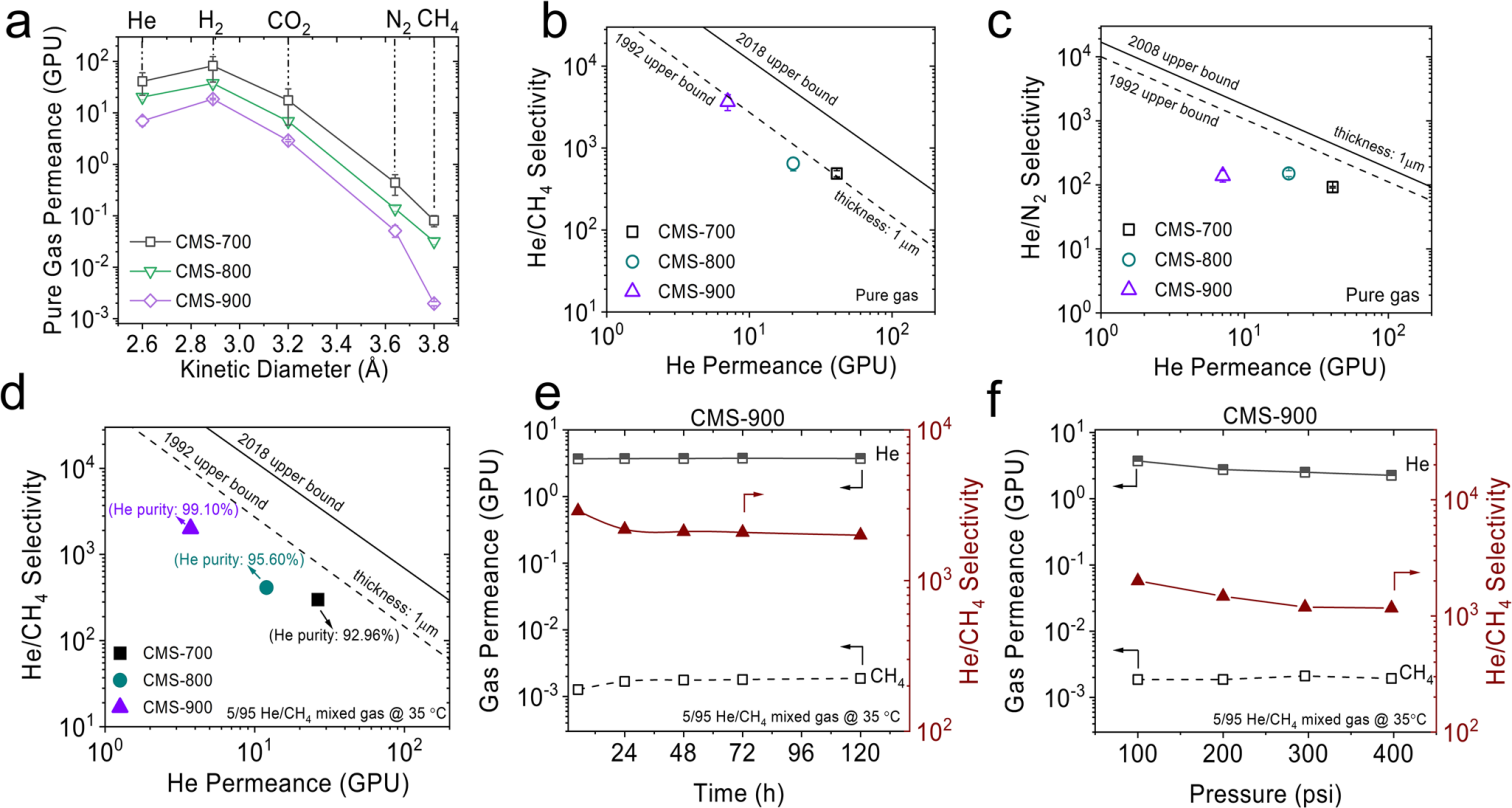
(a) Pure‐gas permeance of CMS fibers pyrolyzed at different temperatures; (b) ideal He/CH_4_ selectivity; (c) ideal He/N_2_ selectivity. (d) He/CH_4_ selectivity under a 5/95 He/CH_4_ mixed‐gas feed (35°C, 100 psia). (e) Long‐term He/CH_4_ separation performance of CMS‐900 under a 5/95 He/CH_4_ feed (35°C, 100 psia). (f) He/CH_4_ separation performance of CMS‐900 under different mixed gas feed pressures.

Figure 2d shows a strong tradeoff between He/CH_4_ selectivity and permeance for the CMS‐900 versus CMS‐800 case. In any case, higher He/CH_4_ selectivity enabled higher helium purity in the permeate stream, which increased from 92.96% to 99.10% as the pyrolysis temperature increased from 700 to 900°C. Long‐term measurements under the 5/95 He/CH_4_ mixed gas feed (Figure 2e) showed that the CMS‐900 fibers stabilize after 48 h and maintained stable performance over 5 days, with a stable He permeance of 3.7 GPU and a He/CH_4_ selectivity exceeding 2000. The effect of feed pressure was also examined, and Figure 2f shows that both He permeance and He/CH_4_ selectivity decreased with increasing pressure, which can be attributed to competitive sorption of CH_4_ over He. Nevertheless, a permeate helium purity of 98.4% was still achieved at 400 psia. These results demonstrate that optimization of the pyrolysis temperature is an effective strategy to tighten ultramicropores in CMS membranes for improving He/CH_4_ selectivity.

This fact notwithstanding, thermally induced ultramicropore tightening at high pyrolysis temperatures is unavoidably accompanied by a decrease in He permeance. To further enhance He/CH_4_ selectivity while maintaining high He permeance, we therefore sought to primarily tighten the larger ultramicropores at the upper end of the pore‐size distribution (Figure 3a). To this end, a post‐pyrolysis hyperaging treatment was applied to CMS fibers. Our previous work showed that freshly prepared CMS membranes undergo self‐retarding physical aging associated with tightening of these larger ultramicropores [24, 25], thereby allowing this process to be tuned by hyperaging [26]. We illustrate this pore‐tuning approach to selectively tailor the larger ultramicropores for He/CH_4_ separation of CMS‐700 fibers by hyperaging in air at different temperatures and durations. As shown in Figure 3b, compared with the pristine CMS‐700 fibers, the hyperaged CMS‐700 fibers exhibited markedly reduced permeance for CO_2_, N_2_, and CH_4_, while the permeance for the smaller He and H_2_ molecules remained nearly unchanged. Consistent with this effect, Figure 3c shows that hyperaging significantly enhanced the He/CH_4_ selectivity with little loss in He permeance. Notably, compared with standard CMS‐900 fibers and other advanced membranes shown in Figure S3 and Table S3, the CMS‐700 fibers hyperaged at 200°C for 1 h achieved more than an order‐of‐magnitude higher pure He permeance (32.6 GPU), with only a slightly lower ideal He/CH_4_ selectivity (2745.6 vs. 3674.4 for CMS‐900 in Figure 3c). A similar tendency was observed for H_2_/CH_4_ separation (Figure S4). Moreover, Figures 3d and S5 indicate that hyperaging also significantly improved He/N_2_ and H_2_/N_2_ separations, and the hyperaged CMS‐700 fibers at 200°C for 1 h showed both substantially higher He permeance and higher He/N_2_ selectivity (568.5) than the CMS‐900 fibers. By contrast, Figure S6 shows that hyperaging did not benefit CO_2_/CH_4_ or N_2_/CH_4_ separations, where the hyperaging treatment led to a noticeable decrease in gas permeance while the CO_2_/CH_4_ and N_2_/CH_4_ selectivities were not improved. These results suggest that hyperaging preferentially improves separations involving small penetrants (He, H_2_) over larger species (CO_2_, N_2_, CH_4_), which is consistent with our hypothesis that hyperaging can selectively tighten the upper end of the ultramicropore distribution [24]. Furthermore, cross‐sectional SEM images of CMS fiber walls in Figure S7 showed no significant differences in overall morphology or skin‐layer thickness before and after hyperaging treatment, indicating that hyperaging primarily affects subtle ultramicropore structure within the dense skin layer rather than inducing visible morphological changes.

**FIGURE 3:**
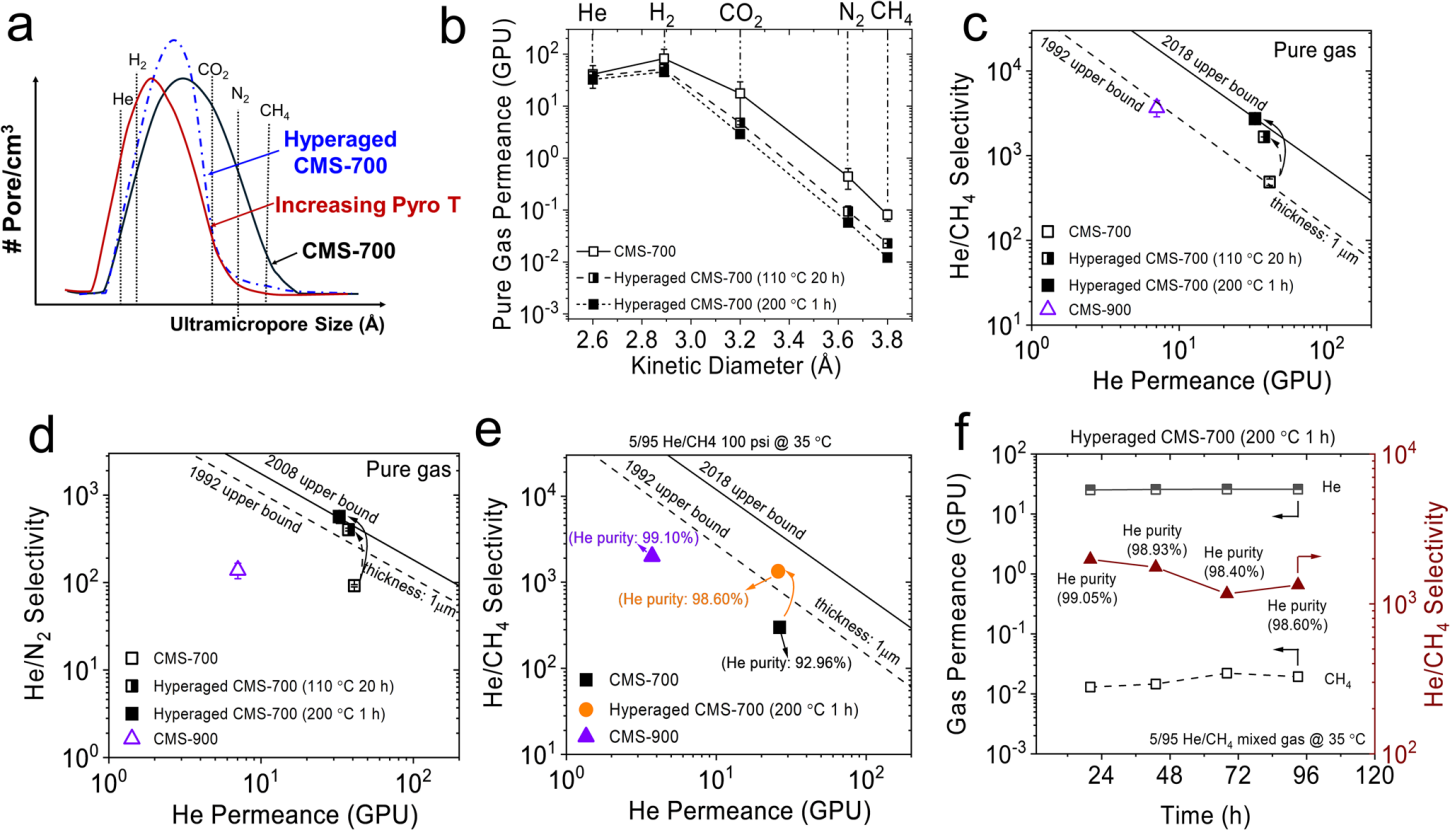
(a) Schematic of ultramicropore tuning by pyrolysis temperature and hyperaging; pure‐gas permeance (b), ideal He/CH_4_ (c) and ideal He/N_2_ (d) selectivity of hyperaged CMS‐700 fibers; (e) comparison of mixed He/CH_4_ separation performance of CMS fibers (5/95 He/CH_4_, 35°C, 100 psia) and (f) Long‐term mixed He/CH_4_ separation performance of hyperaged CMS‐700 fibers (200°C for 1 h).

We also evaluated the separation performance of the hyperaged CMS‐700 fibers (200°C, 1 h) using a 5/95 He/CH_4_ feed at 35°C and 100 psia. As shown in Figure 3e,f, the hyperaged fibers exhibited a clear increase in He/CH_4_ selectivity (1338.1) while retaining high He permeance (about 26 GPU) well beyond the simple 900°C pyrolysis sample. These results confirm that post‐pyrolysis hyperaging provides a more favorable trade‐off between He/CH_4_ selectivity and He permeance than simply increasing the pyrolysis temperature.

Beyond intrinsic material tuning, the practical He productivity of a hollow‐fiber module depends strongly on the amount of membrane area that can be packed into the module shell. When the permeance remains unchanged, the module‐averaged productivity increases with higher packing density. Since the packable membrane area per unit module volume scales inversely with fiber outer diameter (OD) (∝ 1/OD) [27, 28], reducing the CMS fiber OD is an efficient means to increase the membrane area per unit volume (Figure 4a) and thereby maximize He throughput. Thus, besides selectively tightening ultramicropores through hyperaging, engineering CMS hollow fibers with reduced OD represents a complementary and equally critical design lever for achieving high‐performance He/CH_4_ separation at the module level [29].

**FIGURE 4:**
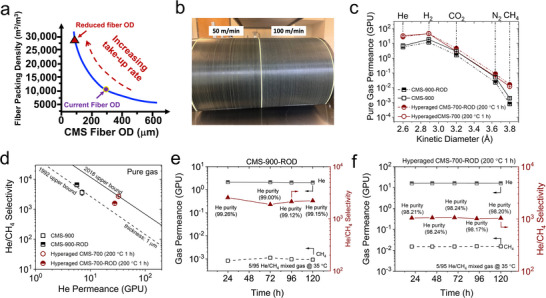
(a) Schematic of fiber packing density as function of OD of CMS fibers; (b) photo of Matrimid hollow fiber precursors spun with take up rate of 50 and 100 m min^−^^1^; pure‐gas permeance (c) and ideal He/CH_4_ (d) and ideal He/N_2_ (d) selectivity of CMS fibers with reduced OD; Long‐term mixed He/CH_4_ separation performance of CMS‐900‐ROD (e) and hyperaged CMS‐700‐ROD fibers (f).

To explore this option, Matrimid hollow fibers with reduced OD were produced by increasing the take‐up rate while holding all other spinning parameters constant. As shown in Figure 4b and Table S2, defect‐free fibers were successfully spun at a take‐up rate as high as 100 m min^−^^1^. SEM images (Figure S8) show that the precursor OD decreased from 323 µm to 121 µm as the take‐up rate increased from 30 to 100 m min^−^^1^; correspondingly, the OD of the resulting CMS‐900 fibers derived from the 100 m min^−^^1^ precursors decreased to 85 µm. Bend tests as shown in Figure S9 indicate that the CMS‐900 fibers with reduced OD (i.e., ROD) remain highly flexible, demonstrating mechanical robustness for module fabrication and practical use. Pure gas permeance was evaluated for CMS fibers with reduced OD (take‐up rate 100 m min^−^^1^), including CMS‐900‐ROD and hyperaged CMS‐700‐ROD, and benchmarked them against CMS fibers prepared from precursors spun at 50 m·min^−^^1^. As shown in Figure 4c,d, reducing OD did not significantly affect gas permeance or He/CH_4_ selectivity. Under a 5/95 He/CH_4_ mixed‐gas feed, both CMS‐900‐ROD and hyperaged CMS‐700‐ROD exhibited stable separation performance (Figure 4e,f). Although CMS‐900‐ROD shows reduced He permeance under mixed‐gas conditions compared with pure gas due to He/CH_4_ competition, the mixed‐gas selectivity remains high and stable. Specifically, CMS‐900‐ROD (OD = 85 µm) showed a stable He permeance of 2.1 GPU and He/CH_4_ selectivity of 2216, yielding permeate helium purity above 99%, comparable to that of CMS‐ 900 fibers with OD of 187 µm (He permeance of 3.7 GPU with He/CH_4_ selectivity of about 2000, as shown in Figure 2e). In comparison, CMS‐700‐ROD (OD < 100 µm) exhibited a higher and stable He permeance (∼16.2 GPU) while maintaining mixed gas He/CH_4_ selectivity above 1000. Notably, the hyperaged CMS‐700‐ROD membranes maintained stable He permeance and He/CH_4_ selectivity for over 120 h (Figure 4f), demonstrating excellent operational stability for practical separation processes. Collectively, these results show that OD reduction increases packable area without significantly compromising fiber mechanics or intrinsic membrane selectivity, thereby enabling higher module‐level He productivity with maintained high He/CH_4_ selectivity. Although reducing fiber diameter requires careful management of bore‐side pressure drop and wall porosity, our results demonstrate that this can be balanced in practice.

In conclusion, we demonstrate a scalable strategy for ultraselective He/CH_4_ separation based on Matrimid‐derived asymmetric CMS hollow fibers, where co‐engineering of ultramicropores and fiber geometry enables high He/CH_4_ selectivity with elevated module‐level He productivity. This combination of mechanically robust hollow‐fiber form, precise ultramicropore engineering, and module‐focused design enables a practical, reproducible platform for industrial helium recovery and can be readily adapted to other challenging small/large gas‐pair separations.

## Conflicts of Interest

The authors declare no conflict of interest.

## Supporting information




**Supporting File**: anie71903‐sup‐0001‐SuppMat.docx.

## Data Availability

The data that support the findings of this study are available from the corresponding author upon reasonable request.
