# Supplementary material for: Matrimid‐Derived Asymmetric Carbon Molecular Sieve Hollow Fibers With Engineered Ultramicropores for Precise Helium Separation
Source: Angew Chem Int Ed Engl. 2026 Mar 23;65(18):e6271309. doi: 10.1002/anie.6271309 (PMC13110757; doi:10.1002/anie.6271309)
Supplement: Supplementary file 1 — Supporting File: anie71903‐sup‐0001‐SuppMat.docx. [file ANIE-65-e6271309-s001.docx]

Supporting Information
©Wiley-VCH 2019
69451 Weinheim, Germany

**Matrimid-Derived Asymmetric Carbon Molecular Sieve Hollow Fibers with Engineered Ultramicropores for Precise Helium Separation**

Zhongyun Liu^a,b^, Yuhe Cao^a,c^, Ryan P. Lively^a^, William J. Koros^a^*

[a] Dr. Z. Liu, Y. Cao, R. P. Lively, W. J. Koros
School of Chemical & Biomolecular Engineering,
Georgia Institute of Technology
311 Ferst Drive, Atlanta, GA 30332, USA
E-mail: [wjk@chbe.gatech.edu](mailto:wjk@chbe.gatech.edu) (W.J.K)

[b] Dr. Z. Liu
Key Lab of Functional Polymers for Sustainability of Jiangsu
School of Energy and Environment
Southeast University, Nanjing, Jiangsu, P.R. China

[c] Dr. Y. Cao

Department of Civil, Environmental and Construction Engineering

The University of Texas at El Paso, El Paso, TX 79968, USA

**Abstract:** Helium/methane (He/CH₄) separation is of strategic importance for energy and industrial applications, yet it remains technically challenging due to the need to simultaneously achieve ultrahigh selectivity and helium productivity. In this work, we report ultraselective Matrimid-derived asymmetric carbon molecular sieve (CMS) hollow fibers in which ultramicropore and fiber geometry are deliberately co-engineered to enable precise He/CH₄ separation with high He productivity at the module level. We showed pyrolysis temperature tuning tightens ångström-scale ultramicropores and enhanced He/CH₄ discrimination; while a targeted post-pyrolysis hyperaging enables selective refining of the ultramicropores; thereby offering high He permeance with exceptional He/CH₄ selectivity. For a 5/95 He/CH₄ mixed gas feed, the hyperaged CMS-700 hollow fibers achieve permeate helium purities of up to 98.6% with He/CH₄ selectivities exceeding 1300 and a stable He permeance of approximately 26 GPU. Beyond this material achievement, fiber geometry optimization through reduction of the outer diameter was achieved to increase the packable membrane area without compromising mechanical integrity or intrinsic separation performance, leading to enhanced module-level He productivity. This integrated co-engineering strategy provides an energy-efficient and industrially viable platform for He recovery and is readily extendable to other challenging small/large gas-pair separations.

**DOI: 10.1002/anie.2016XXXXX**

Table of Contents

1. Experimental Procedures •••••••••••••••••••••••••••••••••••••••••••••••••••••••••••••••••••••••••••••••••••••••••••••••••3-5
2. Supplemental Figures S1-S9 •••••••••••••••••••••••••••••••••••••••••••••••••••••••••••••••••••••••••••••••••••••••••••••6-9
3. Supplemental Table S3 •••••••••••••••••••••••••••••••••••••••••••••••••••••••••••••••••••••••••••••••••••••••••••••••••••••10-11
4. Supplemental References •••••••••••••••••••••••••••••••••••••••••••••••••••••••••••••••••••••••••••••••••••••••••••••••••11
5. Author Contributions •••••••••••••••••••••••••••••••••••••••••••••••••••••••••••••••••••••••••••••••••••••••••••••••••12

1. Experimental Procedures

1.1 Materials

The chemicals used in this work including sure-seal bottles of tetrahydrofuran (THF), anhydrous ethanol, vinyltrimethoxysilane (VTMS) (97% purity), hexane and 1-methyl-2-pyrrolidinone (NMP) were purchased from Sigma-Aldrich and used as received. Anhydrous methanol (20 L) and hexane (20 L) were purchased from BDH Chemicals Co for solvent exchange of hollow fibers. Commercial Matrimid^®^ 5218 polyimide (weight-average molecular weight (Mw) of 71,200 g·mol⁻¹ with polydispersity index (PDI) of 3.6) was obtained from Huntsman International LLC. for fiber spunning. Ultra-high-purity (UHP) argon, helium, hydrogen, carbon dioxide, nitrogen, methane, and 5/95 He/CH₄ mixed gas cylinders were supplied by Airgas for pyrolysis process and gas permeation measurements. Fittings and tubing for module fabrication were obtained from Swagelok^®^ (Georgia).

**1.2 Matrimid hollow fiber spinning**

Matrimid hollow fiber precursors were spun following the spinning procedures and equipment described in our earlier publications^[^[^1^](#_ENREF_1)^]^. Matrimid^®^ 5218 powder was dried in a vacuum oven at 110 °C overnight to remove moisture. The spinning dope was prepared in a sealed glass bottle and mixed on a roller at room temperature until homogeneous. A dope formulation contained 26.2 wt% Matrimid, 53.0 wt% NMP, 14.9 wt% ethanol, and 5.9 wt% THF. The dope was transferred to a 500 mL ISCO syringe pump, degassed overnight at 60 °C prior to spinning. The bore fluid of 92.0 wt% NMP and 8.0 wt% water was loaded in a separate 500 mL syringe pump. Dope and bore fluid were co-extruded through a concentric spinneret under 60 °C and detailed spinning parameters are given in Table S1. After spinning, the fibers were soaked in DI water baths for 3 days to remove the traces of solvent. Then solvent exchange was performed sequentially in methanol (three × 20 min) and hexane (three × 20 min), followed by drying at 75 °C for 3 h in vacuum oven.

To verify that the Matrimid hollow-fiber precursors were defect-free, replicate modules containing 3–4 fibers were assembled and tested. O₂/N₂ permeance and selectivity were measured at 35 °C with bore-side feed at ~50 psig and shell-side permeate at atmospheric pressure. The O_2_ permeance and O₂/N₂ selectivity of prepared Matrimid fiber precursors with different take up rates are given in Table S2.

**Table S1.** Spinning parameters for Matrimid hollow fibers

| **Spinning parameters** | **Values** |
| --- | --- |
| Dope Extrusion Rate | 240 mL/h |
| Bore Fluid Extrusion Rate | 80 mL/h |
| Spinneret Temperature | 60 ℃ |
| Air Gap | 10 cm |
| Quench Bath Temp. | 50 ℃ |
| Take-up Rate | 30, 50 and 100 m/min |
| Bore fluid (NMP/H_2_O) | 92/8 |

**Table S2**. O_2_ permeance and O₂/N₂ selectivity of prepared Matrimid fiber precursors with different take up rates

| **Matrimid fiber precursors** | **O_2_ permeance** | **O₂/N₂ selectivity** |
| --- | --- | --- |
| Take up rate of 30 m min^-1^ | 15.1 GPU | 8.5 |
| Take up rate of 50 m min^-1^ | 15.6 GPU | 7.2 |
| Take up rate of 100 m min^-1^ | 13.8 GPU | 6.0 |

**1.3 Vinyltrimethoxysilane (VTMS) treatment, CMS hollow fibers preparation and hyperaging treatment**

Prior to pyrolysis, VTMS treatment was performed to prevent collapse of the porous support of Matrimid hollow fibers. Briefly, Matrimid fibers were immersed in 10 wt% vinyltrimethoxysilane (VTMS) in hexane for 24 h at 25 °C. The VTMS-saturated fibers were removed, briefly drained, and exposed to ambient moisture at 25 °C for ~24 h to promote hydrolysis/condensation and formation of a crosslinked silica network within the substructure. Fibers were then dried under vacuum at 150 °C for 12 h to remove residual VTMS and moisture.

To prepare CMS hollow fibers, VTMS-treated precursors were placed on a stainless-steel wire mesh inside a quartz tube and carbonized in a three-zone tube furnace under ultra-high-purity (UHP) Ar, following a standard protocol. The tube ends were sealed with metal flanges and silicone O-rings to ensure leak-tightness and pre-purged with UHP Ar (500 sccm) for more than 6 h to reduce O₂ to below 1 ppm. The heating profile was: 50→250 °C at 13.3 °C min⁻¹; 250→585 °C at 3.85 °C min⁻¹; then to the target temperature (700, 800, or 900 °C) at 0.25 °C min⁻¹. Samples were held at the final temperature for 2 h and cooled naturally to room temperature under Ar atmosphere. The resulting CMS fibers were collected for module fabrication and characterization. For hyperaging treatment, freshly made CMS fibers were placed on aluminum foil and thermally treated in air at the specified temperature and duration in a preheated conventional oven.

**1.4 Characterizations**

Scanning electron microscopy (SEM; Hitachi SU8230) was used to characterize the morphologies of Matrimid fiber precursors and CMS fibers. For polymer samples, fibers were soaked in hexane, cryo-fractured in liquid nitrogen with tweezers to preserve the cross-section, and sputter-coated with Au (Hummer 6 Gold/Palladium Sputterer) prior to imaging. For CMS fibers, samples were prepared in air without hexane or liquid-nitrogen treatment and were imaged without conductive coating.

**1.5 Gas permeation tests for CMS hollow fibers**

For gas permeation tests, CMS hollow fibers were potted using lab-scale modules, following procedures described in previous works.^[^[^2^](#_ENREF_2)^]^ To evaluate whether reducing the outer diameter (OD) of a single CMS hollow fiber affects its intrinsic separation performance, all CMS modules in this study were fabricated as single-fiber modules. The separation performance of reduced-OD fibers was directly compared with that of standard-OD fibers under identical testing conditions.

Single-fiber modules were prepared using 3M™ DP-100 epoxy. To ensure complete epoxy curing, the modules were tested 18 hours after being potted. During the 18-hour curing, the connections in the shell side of the CMS module were sealed with parafilm, while the connections in the bore side of the CMS module were kept open to the atmosphere. Subsequently, pure gas permeation measurements were conducted using a variable-pressure, constant-volume permeation system at 35 °C, with an upstream pure gas pressure of 100 psia and a downstream vacuum. For each condition, at least 2-3 independently prepared modules were tested to ensure reproducibility of the results.

For 5/95 He/CH_4_ mixed gas test, a cross-flow configuration was used on the upstream side to minimize concentration polarization, while the constant flow on the upstream was controlled by a stainless-steel flow metering valve (Swagelok, SS-SS2-VH). The stage cut, which equals the flow rate ratio of permeate to feed, was kept at ≤ 1%. After reaching equilibrium, the change in downstream pressure over time was monitored using LabView software. The gas composition in the downstream was determined using a gas chromatograph (Varian 450-GC). The permeance (P_i_/) of the CMS fibers for mixed gas is calculated using equation 1:

$P_{i}=\frac{(\frac{\Delta p}{\Delta t})\times y_{i}\times V_{down}}{A\times R\times T\times(x_{i}\times f_{upstream}-y_{i}\times f_{downstream})}$ (Eq. 1)

where *∆p/∆t* is the effective downstream pressure rise rate during permeation test, which is calculated by deducting the leak rate of setup using the slope of the downstream pressure *vs.* time during permeation; *V_down_* is the downstream volume of permeation system; *A* is the membrane area; R is the gas constant; *T* is the testing temperature; *y_i_* is the mole fraction of component (i) in the downstream, *x_i_* is the mole fraction of component (i) in the feed upstream, *f_upstream_* and *f_downstream_* are the fugacities of the feed upstream and downstream, respectively, which is determined using a Peng-Robinson equation of state and the SUPERTRAPP program developed by NIST.

The perm-selectivity (α_ij_) is calculated by the ratio of the permeability of component i to the permeability of component j (Eq. 2):

$\alpha_{i j}=\frac{P_{i}}{P_{j}}$ (Eq. 2)


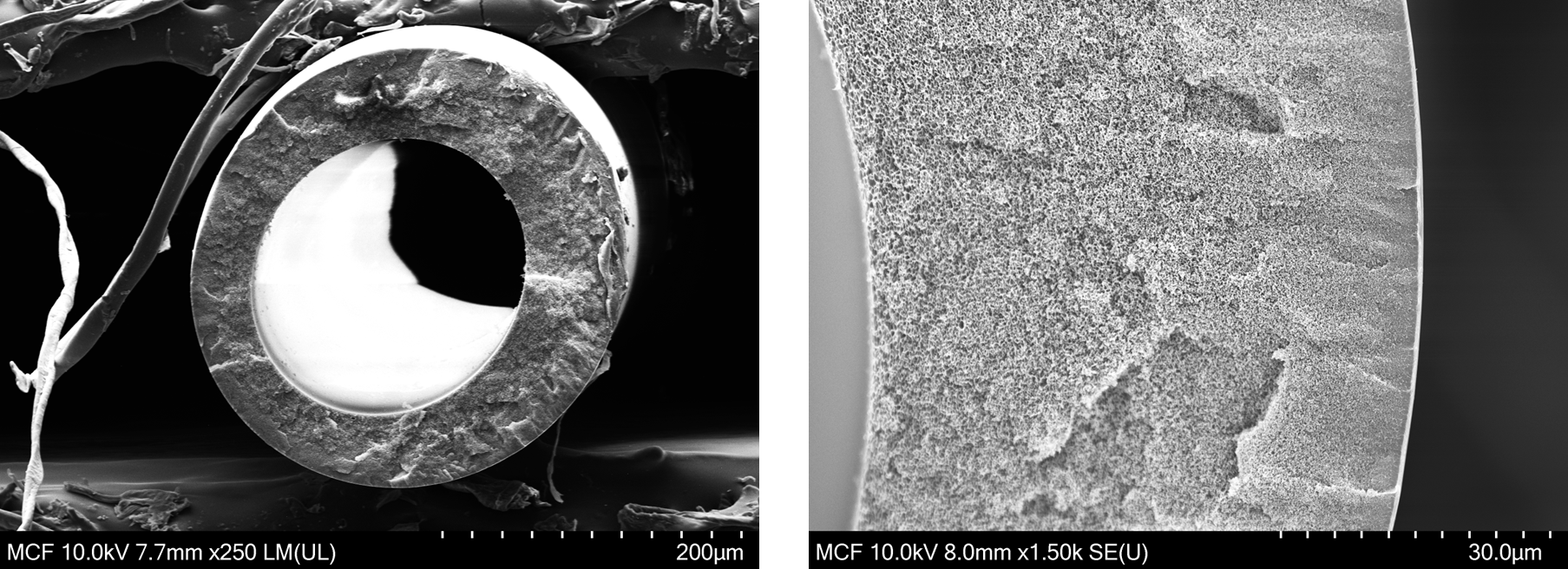


**Figure S1**. SEM images of Matrimid hollow fiber with take up rate of 50 m min^-1^


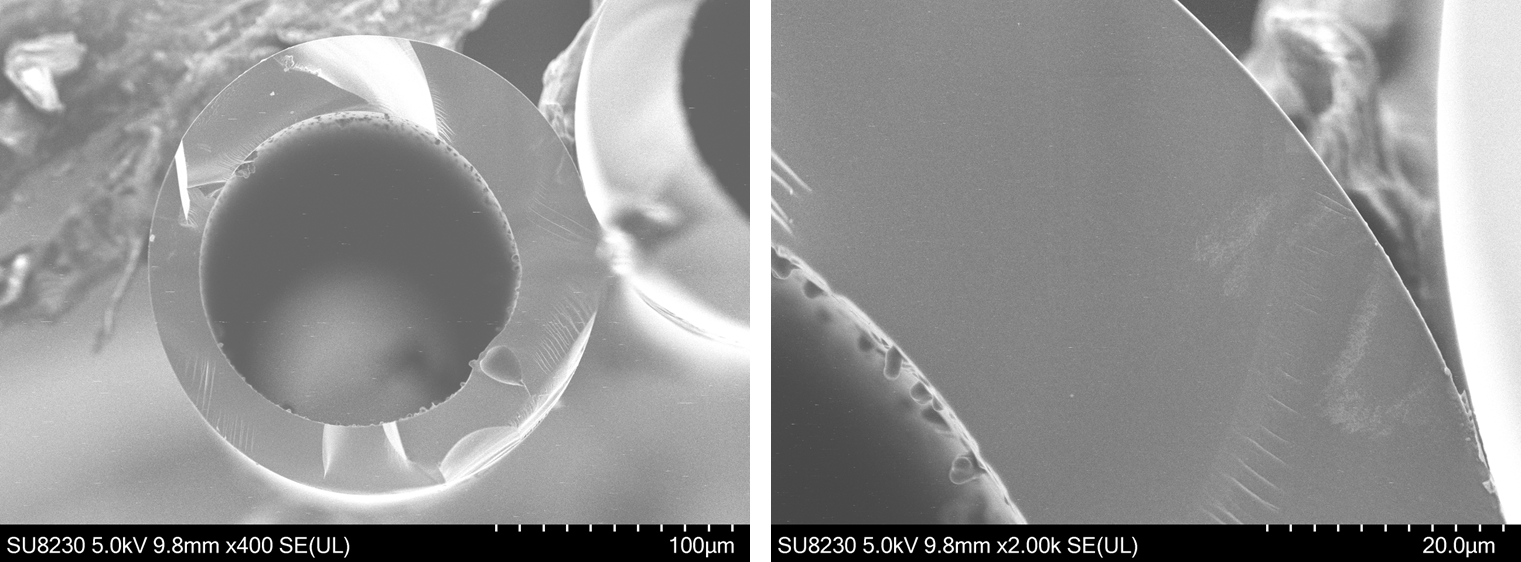


**Figure S2.** SEM images of CMS-900 hollow fiber without VTMS


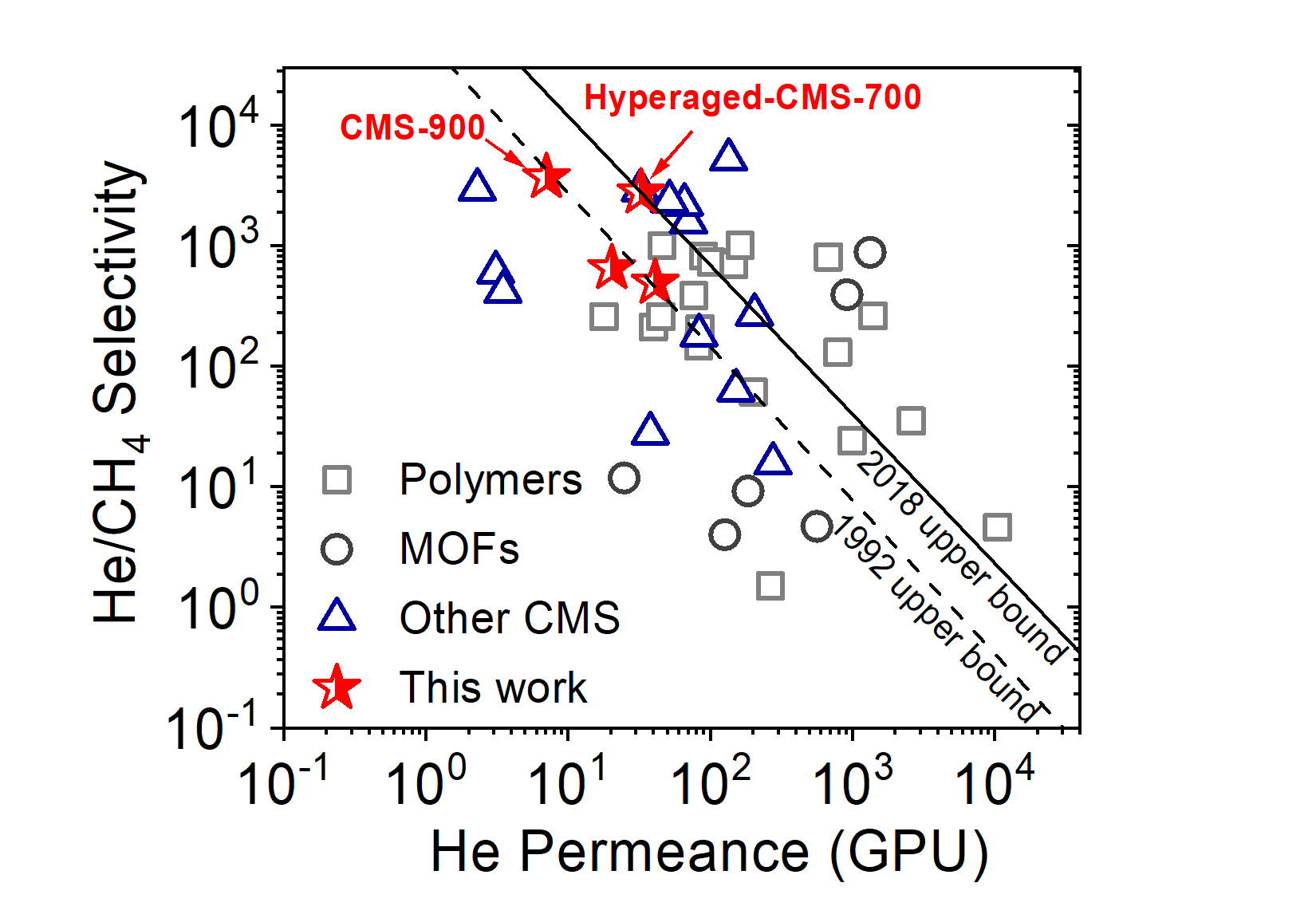


**Figure S3.** Comparison of He/CH_4_ separation performance of Matrimid-derived CMS hollow fibers with reported other polymer/CMS hollow fibers and MOFs TFC membranes (detailed data is provided in Table S3).


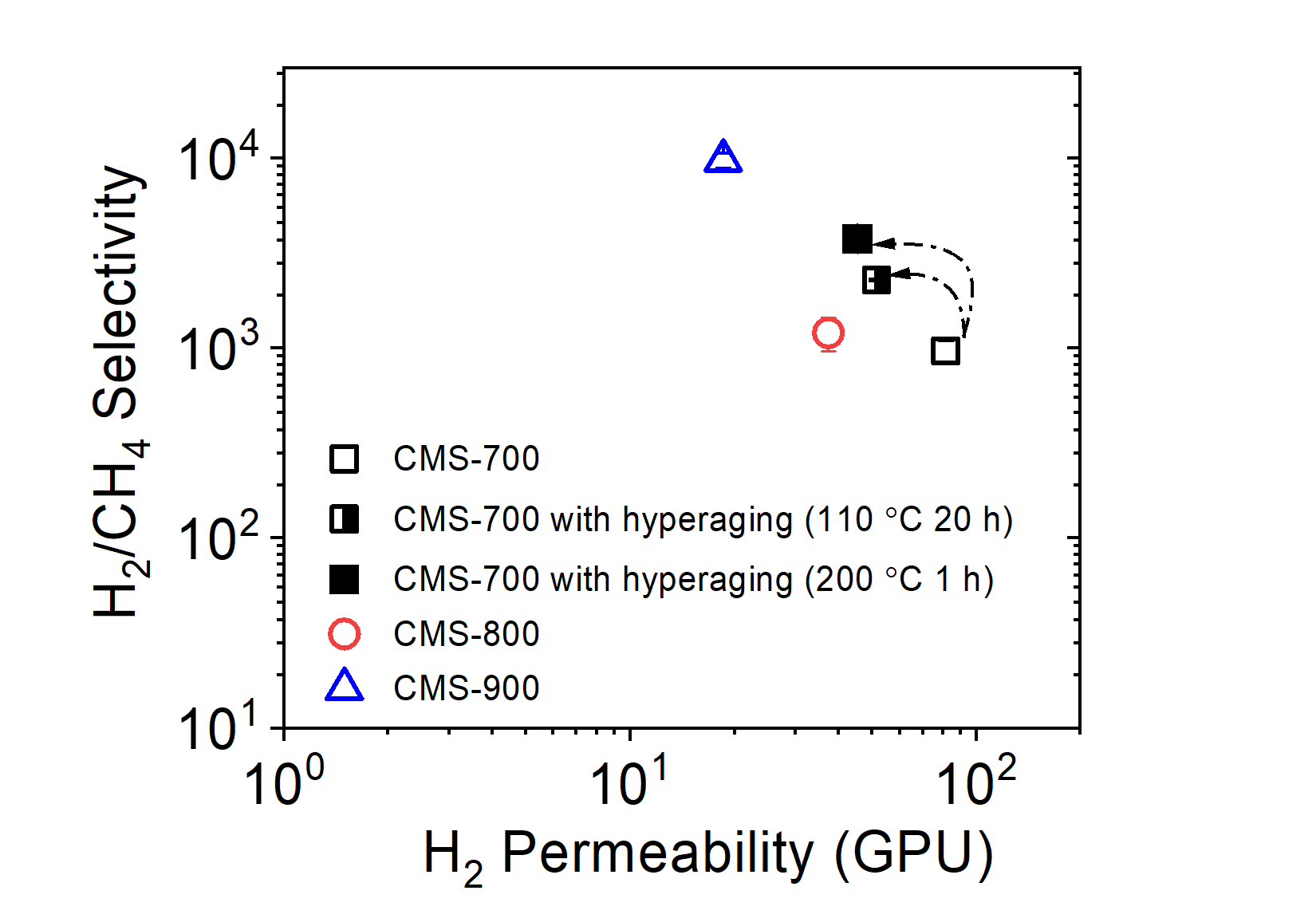


**Figure S4.** Comparison of He permeance and ideal He/CH_4_ selectivity of hyperaged CMS-700 fibers with CMS fibers pyrolyzed under different temperatures


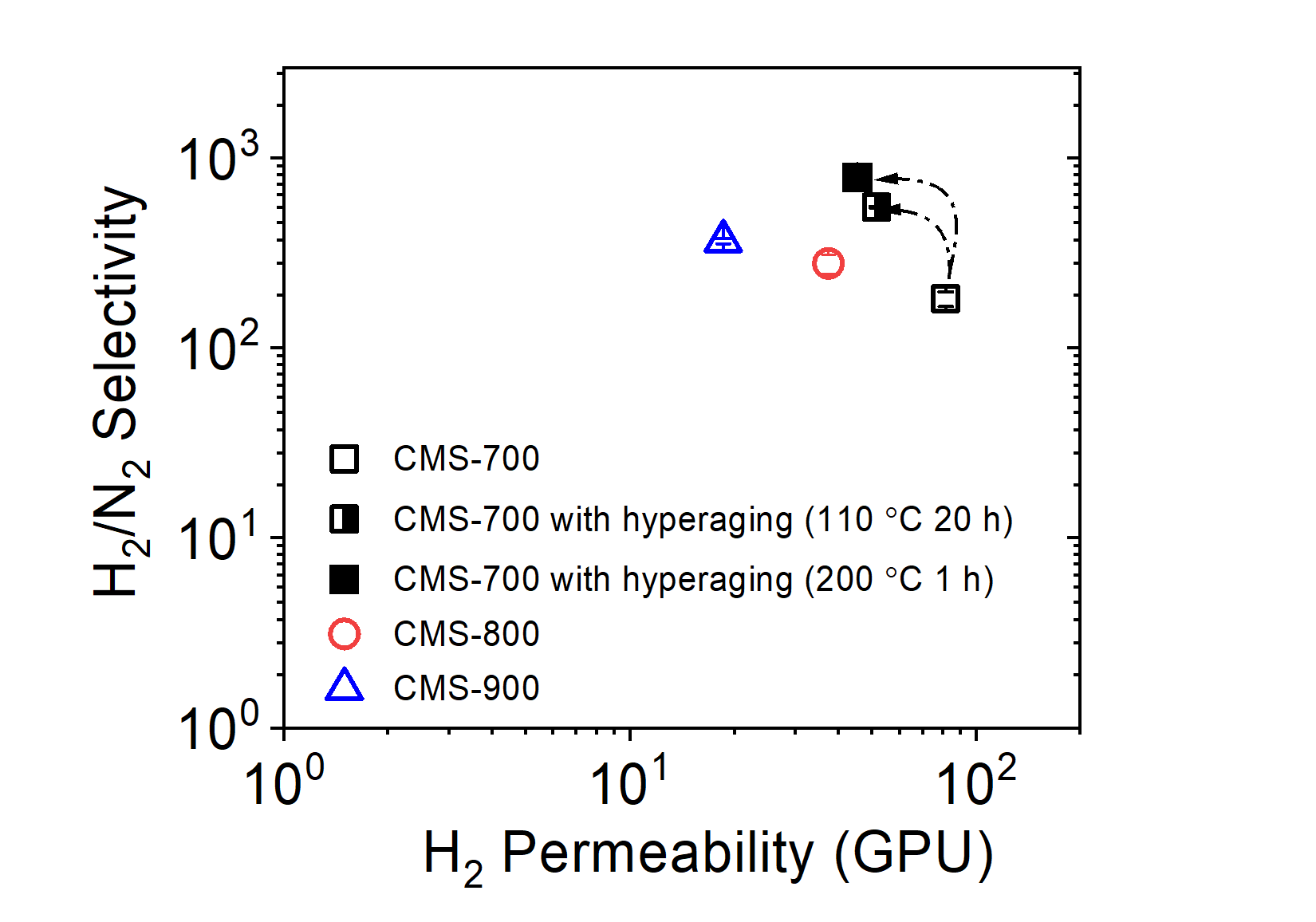


**Figure S5.** Comparison of H_2_ permeance and ideal H_2_/N₂ selectivity of hyperaged CMS-700 fibers with CMS fibers pyrolyzed under different temperatures

**Figure S6.** Hyperaging effects on CO_2_/CH_4_ and N_2_/CH_4_ separation performance (single-gas permeance and corresponding ideal selectivity) of CMS fibers


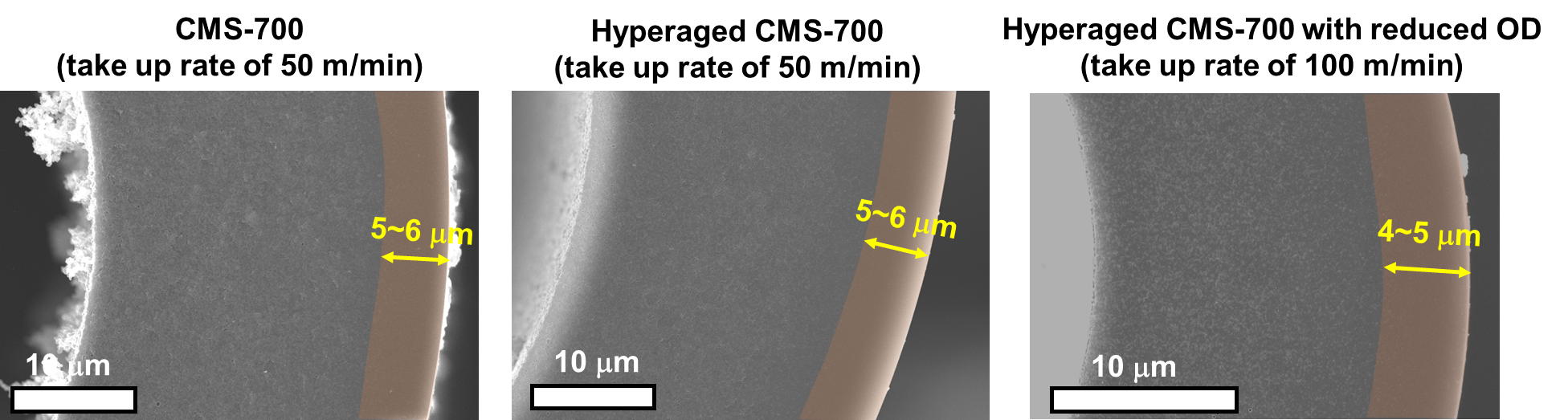


**Figure S7.** Cross-sectional SEM images of CMS-700 hollow fiber walls illustrating the effects of hyperaging on CMS with different OD. The shading shown illustrates a similar region to that in Fig. 1b for CMS 700.


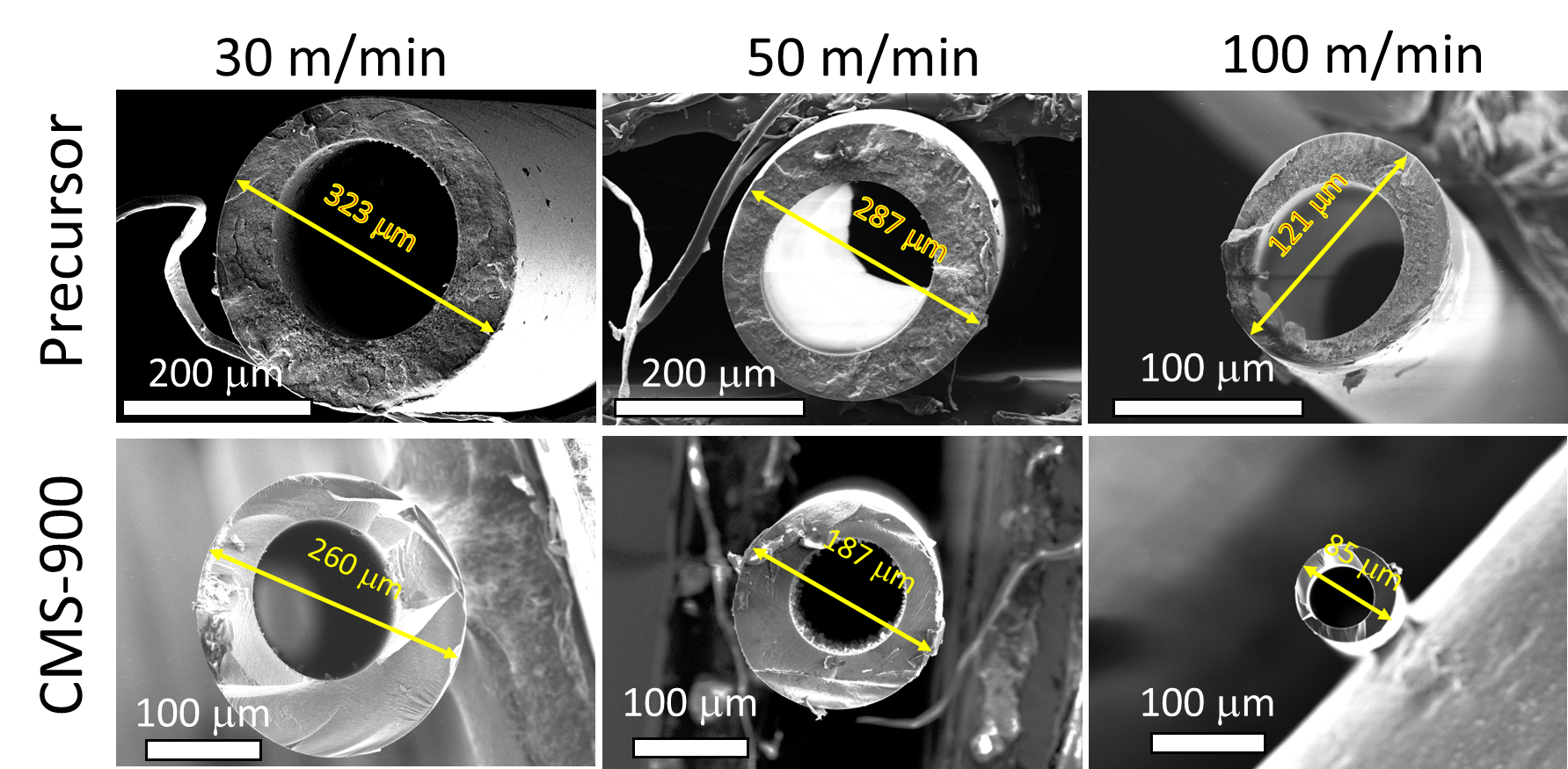


**Figure S8.** SEM images of Matrimid hollow fiber precursors and CMS-900 fibers with various take up rates from 30 m min^-1^ to 100 m min^-1^.


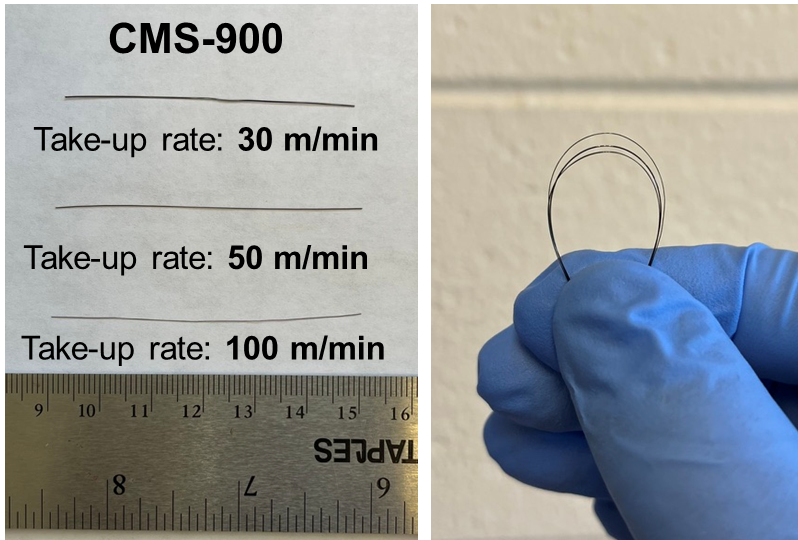


**Figure S9.** Bend test of CMS-900 fibers

**Table S3.** Comparison of He/CH_4_ separation performance of Matrimid-derived CMS hollow fibers with reported other polymer/CMS hollow fibers and MOFs TFC membranes.

| **Membrane** | | **Test conditions** | **He Permeance (GPU)** | **He/CH_4_ Selectivity** | **Ref.** |
| --- | --- | --- | --- | --- | --- |
| Polymer membranes | 6FDA_0.9_-ODPA_0.1_-mPDA hollow fiber | 35 °C, pure gas | 85.0 | 204.0 | ^[^[^3^](#_ENREF_4)^]^ |
|  | Polybenzimidazole on Porous Supports | N/A, pure gas | 46 | 1000 | ^[^[^4^](#_ENREF_5)^]^ |
|  | TR-X1-F0 hollow fiber  (TR-PBOI) | 35 °C, pure gas | 996 | 24 | ^[^[^5^](#_ENREF_6)^]^ |
|  | TR-X1-F1 hollow fiber  (TR-PBOI) | 35 °C, pure gas | 678 | 800 |  |
|  | PIM-1-F0 hollow fiber | 35 °C, pure gas | 265 | 1.5 |  |
|  | PIM-1-F5 hollow fiber | 35 °C, pure gas | 202 | 61 |  |
|  | Matrimid hollow fiber | 20-23 °C, pure gas | 83.2 | 146 | ^[^[^6^](#_ENREF_7)^]^ |
|  | Fluorinated Matrimid hollow fiber  (2 min) | 20-23 °C, pure gas | 77.0 | 385 |  |
|  | Fluorinated Matrimid hollow fiber  (265 min) | 20-23 °C, pure gas | 89.7 | 815 |  |
|  | Matrimid-Cyclen-5% (1.8 mm)  TFC membrane | 25 °C, pure gas | 102 | 728 | ^[^[^7^](#_ENREF_8)^]^ |
|  | Teflon® AF 2400 TFC membrane  (perfluoropolymers) | 22 °C, pure gas | 10500 | 4.6 | ^[^[^8^](#_ENREF_9)^]^ |
|  | Hyflon AD60 TFC membrane | 22 °C, pure gas | 2600 | 35 |  |
|  | Cytop TFC membrane | 22 °C, pure gas | 790 | 130 |  |
|  | Copolymer D TFC membrane | 22 °C, pure gas | 1400 | 260 |  |
|  | 6FDA-mPDA_0.65_-DABA_0.3_-TFMB_0.05_ hollow fiber (pristine) | 35 °C, pure gas | ~40 | 210 | ^[^[^9^](#_ENREF_10)^]^ |
|  | 6FDA-mPDA_0.65_-DABA_0.3_-TFMB_0.05_ hollow fiber (crosslinked) | 35 °C, pure gas | ~18 | 258 |  |
|  | 6FDA-mPDA_0.65_-DABA_0.3_-TFMB_0.05_ -cerium (PI–Ce) complex hollow fiber | 35 °C, pure gas | 45 | 257 | ^[^[^10^](#_ENREF_11)^]^ |
|  | Fluorinated PDMS-Matrimid hollow fiber (50W-65Pa-500s) | 35 °C, He/CO_2_/CH_4_  (0.3/5/94.7, v/v/v) at 4 bar | 147 | 697 | ^[^[^11^](#_ENREF_12)^]^ |
|  | Fluorinated PDMS-Matrimid hollow fiber (50W-65Pa-500s) | 35 °C, He/CO_2_/CH_4_  (0.3/5/94.7, v/v/v) at 40 bar | 163 | 1005 |  |
| MOF membranes | ZIF-7 on ZIF-90 | 150 °C, pure gas | 24.8 | 11.8 | ^[^[^12^](#_ENREF_13)^]^ |
|  | ZIF-8 (2.5 mm) | 35 °C, pure gas | 565 | 4.7 | ^[^[^13^](#_ENREF_14)^]^ |
|  | ZIF-8 (80 mm) | 25 °C, pure gas | 126.6 | 4.0 | ^[^[^14^](#_ENREF_15)^]^ |
|  | 3.50% C70@ZIF-8 | 23 °C, pure gas | 185.4 | 9.2 | ^[^[^15^](#_ENREF_16)^]^ |
|  | MTV-ZIF-A_60_C_40_ | 25 °C, 0.6% He/99.4% CH_4_ at 5 bar | 1335 | 876 | ^[^[^16^](#_ENREF_17)^]^ |
|  | MTV-ZIF-A_60_C_40_ | 25 °C, 50% He/50% CH_4_  at 5 bar | 916 | 391 |  |
| CMS membranes | P84-900°C, 30 min | 35 °C, pure gas | 3.1 | 603.9 | ^[^[^17^](#_ENREF_18)^]^ |
|  | P84-900°C, 60 min | 35 °C, pure gas | 2.3 | 2925 |  |
|  | P84-900°C | 60 °C, pure gas | 3.5 | 415.3 | ^[^[^18^](#_ENREF_19)^]^ |
|  | Kapton-He-600 °C | 35 °C, pure gas | 276.3 | 15.6 | ^[^[^19^](#_ENREF_20)^]^ |
|  | Phenolic resin-800 °C | 35 °C, pure gas | 152.2 | 63.0 | ^[^[^20^](#_ENREF_21)^]^ |
|  | PIM-PI/alumina-700 °C | 35 °C, pure gas | 83.69 | 180.75 | ^[^[^21^](#_ENREF_22)^]^ |
|  | PPO on ceramic support-800 °C | 35 °C, pure gas | 204.6 | 268.6 | ^[^[^22^](#_ENREF_23)^]^ |
|  | PVDC-PVC-700 °C | 35 °C, pure gas | 38.0 | 27.8 | ^[^[^23^](#_ENREF_24)^]^ |
|  | 6FDA_0.9_-ODPA_0.1_-mPDA-CMS-550 | 35 °C, pure gas | 70.6 | 1560 | ^[^[^24^](#_ENREF_25)^]^ |
|  | 6FDA_0.9_-ODPA_0.1_-mPDA-CMS-550-F-1min | 35 °C, pure gas | 65.8 | 2193 |  |
|  | 6FDA_0.9_-ODPA_0.1_-mPDA-CMS-650-F-1min | 35 °C, pure gas | 51.6 | 2334 |  |
|  | 6FDA_0.9_-ODPA_0.1_-mPDA-CMS-800-F-1min | 35 °C, pure gas | 32.4 | 2895 |  |
|  | Cellulose derived CMS-850 | 130 °C, pure gas | ~ 135 | ~ 5192 | ^[^[^25^](#_ENREF_26)^]^ |
|  | Matrimid-derived CMS-700 hollow fiber | 35 °C, pure gas | 41 | 488.5 | This work |
|  | Matrimid-derived CMS-800 hollow fiber | 35 °C, pure gas | 20.3 | 646.8 |  |
|  | Matrimid-derived CMS-900 hollow fiber | 35 °C, pure gas | 7.0 | 3674.4 |  |
|  | Matrimid-derived CMS-700 hollow fiber (hyperaged at 200 °C 1 h) | 35 °C, pure gas | 32.6 | 2745.2 |  |
|  | Matrimid-derived CMS-700 hollow fiber (hyperaged at 200 °C 1 h) | 35 °C, 5% He/ 95% CH_4_ | 26.0 | 1338.1 |  |

# References

[1] D. T. Clausi, W. J. Koros, *Journal of Membrane Science* **2000**, *167*, 79–89.

[2] a) P. Arab, Z. Liu, M. Nasser, W. Qiu, M. Martinez, D. Flick, A. Roy, J. Liu, W. J. Koros, *Carbon* **2021**, *184*, 214–222; b) Z. Liu, W. Qiu, W. J. Koros, *Angewandte Chemie International Edition* **2022**, *61*, e202210831.

[3] L. Liu, Q. Wu, S. Wang, W. Lai, P. Zheng, C. Wang, X. Wei, S. Luo, *Industrial & Engineering Chemistry Research* **2022**, *62*, 708–716.

[4] X. Wang, M. Shan, X. Liu, M. Wang, C. M. Doherty, D. Osadchii, F. Kapteijn, *ACS Applied Materials & Interfaces* **2019**, *11*, 20098–20103.

[5] J. G. Seong, W. H. Lee, J. Lee, S. Y. Lee, Y. S. Do, J. Y. Bae, S. J. Moon, C. H. Park, H. J. Jo, J. S. Kim, *Science advances* **2021**, *7*, eabi9062.

[6] D. Syrtsova, A. Kharitonov, V. Teplyakov, G.-H. Koops, *Desalination* **2004**, *163*, 273–279.

[7] W. He, X. Wang, J. Guan, Q. Liang, J. Ma, Y. Liu, H. Zhang, C. Zhang, J. Liu, *Nature Communications* **2026**.

[8] Y. Okamoto, H. Zhang, F. Mikes, Y. Koike, Z. He, T. C. Merkel, *Journal of membrane science* **2014**, *471*, 412–419.

[9] Z. Li, T. Han, W. Lai, J. Ma, Y. Zhang, Q. Wu, C. Wang, C. Liao, S. Luo, *Journal of Membrane Science* **2023**, *688*, 122126.

[10] Z. Li, W. Lai, Y. Sun, T. Han, X. Liu, C. Liao, S. Luo, *Journal of Membrane Science* **2025**, *715*, 123480.

[11] C. Wang, X. Chen, X. Liu, Z. Li, R. Liu, S. Luo, S. Zhang, *Angewandte Chemie International Edition* **2025**, *64*, e202512119.

[12] Z. Liu, Y. Liu, H. Wang, Z. Qu, J. Hou, H. Meng, H. Fan, *Industrial & Engineering Chemistry Research* **2025**, *64*, 6106–6113.

[13] D. Liu, X. Ma, H. Xi, Y. Lin, *Journal of membrane science* **2014**, *451*, 85–93.

[14] N. Hara, M. Yoshimune, H. Negishi, K. Haraya, S. Hara, T. Yamaguchi, *Journal of Membrane Science* **2014**, *450*, 215–223.

[15] J. Han, H. Wu, H. Fan, L. Ding, G. Hai, J. r. Caro, H. Wang, *Journal of the American Chemical Society* **2023**, *145*, 14793–14801.

[16] Y. Liu, T. Li, Z. Fan, W. Lv, Y. Liao, Z. Wang, M. D. Guiver, D. Maspoch, J. Jin, *Journal of the American Chemical Society* **2026**.

[17] E. P. Favvas, N. S. Heliopoulos, S. K. Papageorgiou, A. C. Mitropoulos, G. C. Kapantaidakis, N. K. Kanellopoulos, *Separation and Purification Technology* **2015**, *142*, 176–181.

[18] E. Favvas, E. Kouvelos, G. Romanos, G. Pilatos, A. C. Mitropoulos, N. Kanellopoulos, *Journal of Porous Materials* **2008**, *15*, 625–633.

[19] J. Su, A. C. Lua, *Journal of Membrane Science* **2007**, *305*, 263–270.

[20] P.-S. Lee, D. Kim, S.-E. Nam, R. R. Bhave, *Microporous and Mesoporous Materials* **2016**, *224*, 332–338.

[21] W. Ogieglo, T. Puspasari, X. Ma, I. Pinnau, *Journal of Membrane Science* **2020**, *597*, 117752.

[22] H.-J. Lee, M. Yoshimune, H. Suda, K. Haraya, *Journal of membrane science* **2006**, *279*, 372–379.

[23] T. A. Centeno, A. B. Fuertes, *Carbon* **2000**, *38*, 1067–1073.

[24] Q. Wu, L. Liu, Y. Jiao, Z. Li, J. Bai, X. Ma, S. Luo, S. Zhang, *Angewandte Chemie* **2024**, *136*, e202400688.

[25] L. Lei, F. Pan, A. Lindbråthen, X. Zhang, M. Hillestad, Y. Nie, L. Bai, X. He, M. D. Guiver, *Nature Communications* **2021**, *12*, 268.

# Contributions

Z. Liu and W. J. Koros conceived the idea and designed the experiments. Z. Liu prepared Matrimid hollow fibers and CMS fibers, performed the characterization and gas performance measurement, and wrote the first draft of the manuscript. Y. Cao assisted in permeation measurement and data analyses. R. P. Lively and W. J. Koros guided the work and finalized the manuscript. All authors contributed to writing and revising the manuscript.
